# Lung Function in Childhood: Designing the Future Trajectories

**DOI:** 10.3390/children10061036

**Published:** 2023-06-09

**Authors:** Michele Ghezzi, Ahmad Kantar

**Affiliations:** 1 Pediatric Department, Buzzi Children’s Hospital, 20154 Milan, Italy; 2 Pediatric Asthma and Cough Centre, Gruppo Ospedaliero San Donato, 24036 Bergamo, Italy; kantar@centropediatricotosse.com

Lung development is a highly regulated process that may be disrupted by both genetic and environmental factors beginning at an early age. Throughout childhood and into adult life, substantial heterogeneity exists in the trajectories by which lung function patterns develop. It is clear that many adults with lung disease are actually the targets of life-adverse events that took place long before they had any symptoms of respiratory disease. Lung function evaluation in patients with chronic respiratory disease remains a key tool in the health professional’s toolbox. Lung function testing is indicated not only in overall guidelines for major respiratory diseases such as asthma and cystic fibrosis, but also in rare diseases. Epidemiological studies have revealed the importance of various environmental factors on lung health. Lifetime lung function is related to quality of life and longevity [1].

Increased use of spirometry may result in an increased proportion of patients diagnosed early from a functional point of view and, possibly, in a better management of airway disease during follow-up. Lung function impairment at the physiological peak in early adulthood is associated with adverse health outcomes throughout the life course [2].

Forced expiratory volume in 1 s (FEV1) reaches its maximal level in late adolescence or early adulthood and remains stable for several years, a period known as the plateau of lung function, before gradually declining thereafter.

As we know, spirometry remains the most commonly applied lung function test, and it is used as the gold-standard test for all lung conditions, especially in children. Even though spirometry requires the child’s collaboration and is considered feasible from 6 years of age, pediatric pulmonology departments and trained physicians can obtain acceptable tests from preschool children. Other available lung function tests, such as oscillometry and plethysmography, are considered complementary for obtaining a more analytical representation of lung function. This Special Issue offers diverse practices of lung function monitoring in pediatric pulmonology.

Asthma is the most common chronic airway disease in children. It is very important to monitor therapeutic efficacy in disease control in these young patients. Therapeutic failure and the consequent worsening of lung function increase the risk of severe exacerbations [3]. A lack of disease control substantially impacts the quality of life of the patients and their caregivers [4]. Lung function tests are also useful in identifying mechanisms that predispose a patient to develop asthma, among allergies and skin barrier dysfunction [5].

The increased prevalence of asthma is associated with an increase in obesity. Even though the mechanisms are still unclear, both conditions have a negative impact on lung function. Obesity is associated with the deterioration of lung function [6]. 

Increased interest in children with non-cystic fibrosis bronchiectasis following the development of ERS guidelines has recently been observed [7]. Lung function testing is considered a parameter for evaluating the severity of exacerbation in these patients [8]. It is important to recognize the risk factors and to monitor the follow-up in terms of exacerbation and lung function in these patients, as well as in the subgroup of children with primary ciliary dyskinesia [9,10]. As already evidenced in cystic fibrosis, *Pseudomonas aeruginosa* represents the most relevant risk factor for both exacerbation and lung function worsening [11]. 

Emerging evidence indicates that chronic obstructive pulmonary disease (COPD) can arise from multiple impaired lung function pathways, including those that stem from poor lung function in childhood. 

The efficacy of the new treatment options available for rare and severe diseases such as cystic fibrosis should be evaluated for several criteria, among which lung function is one of the most important. To acquire this information, it is important to apply all the available techniques, such as the lung clearance index [12]. 

The different techniques available also represent an opportunity to evaluate interventions in habits such as prolonged breastfeeding [13]. 

Children with medical complexity represent a growing population who require significant healthcare resources and multidisciplinary care. Long-term ventilation indications and management guidelines are crucial in following patients and their families [14]. Our medical era offers new therapies for various respiratory diseases, including the so-called “orphan disease”. This may lead to reducing the impact of genetic and environmental disorders on lung function and respiratory health [15].
